# Supplementary material for: Fast capillary waves on an underwater superhydrophobic surface
Source: Nat Commun. 2025 Feb 12;16:1568. doi: 10.1038/s41467-025-55907-w (PMC11821838; doi:10.1038/s41467-025-55907-w)
Supplement: Supplementary file 1 — Supplementary Information [file 41467_2025_55907_MOESM1_ESM.pdf]

Supplementary Information for the document entitled:
Fast capillary waves on an underwater superhydrophobic surface

1. SUPPLEMENTARY SECTIONS

A. Details on the wave generation

Our interfacial waves travelling on the plastron are generated by an amplitude modulated (AM) ultrasound (US) signal with acoustic frequency $f_{ac} = 2.5$ MHz and varying AM period in the range 100-500 µs. The generation of this interface perturbation essentially results from the acoustic wave having sufficient time-averaged energy density to provide an acoustic radiation force (ARF) overcoming the resisting forces arising from the local interfacial tensions [1]. Allowed by the gas-filled plastron mechanical compliance and acoustic impedance mismatch with water, the interface deformation is analogous to an acoustic fountain created by focused US [2, 3].

Since the direct observations of the mechanical displacement are challenging from a side view due to optical distortion caused by the micropatterned structure, the capability of ARF to perturbate a gas-water interface is exemplified in Fig. S3 in a gas-filled microchannel (width and height of 75 µm and 100 µm, respectively). In this exemplary case, the displacement of the gas-water interface reaches up to ∼20 % of the plastron thickness. For quantitative spatiotemporal study of the perturbations along the superhydrophobic surface, this work concentrates to studies with top-view perspectives of micropillared array with gas occupying the space between the pillars. However, the side-view results of Fig. S3 allowed to conclude that bright and dark optical information of the top-viewed circular plastronic waves presented in the main document respectively corresponded to concave and convex bulges of the water interface, from the point of view of the camera, placed behind the superhydrophobic surface, as schematised in Fig. 1 (setup). This follows a similar logic as in the work of Walbridge and Woodward [4], where a convex bulge of the water interface, from the point of view, translates into a bright optical information. The interfacial waves, oscillating in the kHz range, start to propagate as a result of the US actuation, with characteristic time scale ranging from tens to hundreds of microseconds, which is a considerably slower event than that of the acoustic time scale oscillatory actuation (period T_{ac} = 0.4 µs at 2.5 MHz). Therefore, this work does not study the rapid acoustic time-scale dynamics of the plastron, but rather the fluidic time-scale phenomena driven by the second-order nonlinear forces generated by the acoustic field.

It should be noted that care has been taken to generate these waves near the center of the superhydrophobic surface, so that these propagating ripples would vanish before reflecting on the edges of the plastron and coming back to interfere with the plastron activity observed in the camera frame. In this way, we were able to consider the plastron as a free-field medium for carrying these superhydrophobic-supported plastronic waves.

Employing a low and fixed signal amplitude $A = 0.5$ V throughout the experimental investigation aimed at limiting the possible nonlinear effects, such as the onset of harmonics in the acoustic signal or the inconstancy of the energy density of the plastronics waves. Together, this allowed to study the waves behaviour as a function of the plastron geometry, as independently as possible from the other experimental parameters, such as the wave amplitude. We believe this is the case, to a limited extent. It is possible that the distance imposed by the main lobe and the first side lobe of the high-intensity focused ultrasound (HIFU) transducer's directivity ($\simeq$ 0.8 mm), as shown in Fig. 1(e), promotes the plastronic waves with wavelength matching that distance. In the earliest steps of this work, by generating the plastronic waves with a short US pulse (20 µs), it interestingly appeared that their wavelength was in the range 650-850 µm, as shown in Fig. S6(a), therefore nearing that 0.8 mm distance. The associated ranges of wave frequency and phase speed are given in Fig. S5 and in Fig. S7(a-b). At a later stage of this work, because a better control of the plastronic waves frequency was desirable to facilitate the results analysis at a given frequency, a driving force modulated in amplitude with a given frequency was preferred over the short pulse configuration. The detectable plastronic waves so-produced by ARF with controlled frequency had a wavelength in the range 500 - 1700 µm. Within this range, it is possible that these waves generated at constant driving voltage do not have a constant amplitude across different wavelength. However, considering that an access to the side-view wave amplitude is not conceivable within the current experimental arrangement, this cannot be

confirmed. The possible variation of wave amplitude was therefore disregarded across all the results with varying wavenumber and plastron geometry.

This paragraph comments on the absence of Cassie-to-Wenzel transition reported in this work, while the employed acoustic pressures are superior than the theoretical critical impalement pressure of the investigated plastrons [5]. While the existing theoretical models consider a static pressure, the acoustic forcing used here is of short duration (< 1 ms) and modulated in amplitude. Possibly, this does not provide sufficient time for the plastron collapse to occur through interface touchdown or sliding of the contact line during a single period of plastronic wave, which are the typically reported mechanisms of wetting [5]. We confirmed experimentally (unpublished data) that a longer actuation with same acoustic parameters can easily collapse the plastron, suggesting that the plastron dynamics is restrained by hydrodynamics phenomena, such as liquid inertia, air flow or dissipation at the contact line, with slow timescale compared to the duration of the US actuation.

B. Details on the wave attenuation

The wave attenuation ratio α, as investigated in this work, comes from the power law $e^{\alpha(f)r}$ and is expressed in dB mm^{-1}. It is calculated based on a spectrogram analysis of the wave optical information, as shown in Fig. S10(f). Because a trend in the attenuation ratio α of the plastronic waves induced by an AM pulse is not clearly emerging, this part of the investigation is limited to the results of the experimentation on the plastronic waves induced by a short, not-modulated ultrasound pulse. Fig. S6(d) shows that the wave attenuation is rather high, in the range 0.6-2 dB mm-1, for all configurations of pillar height. Also, it seems that higher pillars provide the gas phase a good elastic potential, little affected by attenuation, compared to the case of thinner plastrons characterised by a more important attenuation. In the following, three comments on the wave attenuation are provided, and discussion on (i) the influence of the shape of the pillar tips, (ii) the surface contamination and (iii) the meniscus dissipation.

As a consequence of the manufacturing process, the shape of the pillar tips (where the three phases gas, water and PDMS meet) appears sharper for shorter pillars and more round for higher ones, as it can be seen in Fig. S9(k-o). The contact angle that the gas-water interface forms with the solid phase behaves hysterically, meaning that the three-phases contact line will remain pinned, as long as the local gas-water interface does not bend extensively. With the passage of *e.g.*, an interfacial wave, if the gas-water interface moves so much that the interfacial tensions between the three phases cannot hold the line anymore, the contact line may separate and, due to the surface nanometric roughness, advance in a stick-slip motion [6]. This jerking motion is highly energy-consuming, by virtue of frictional energy loss and release of heat. The dynamic system, if there is sufficient energy input, manages to overcome the energy barrier imposed by its pinned contact, which is especially intrinsic to the complexity of the solid surface geometry [7], and reported to be more energy-consuming in the case of an angled surface [8]. By deduction, this energy loss will appear more likely in the case of our 53 and 71 µm high pillars and their rounded tip, than our shorter pillars whose the tip displays a sharper borderline, resulting in possible differences in wave behaviour. This could explain an increased wave damping in the case of higher pillars. A micro-scale study of the contact line and angle dynamics would be yet needed in the future to confirm or infirm this, as a function of the pillar tip shape, with constant pillar height and spacing.

Surface contamination was prevented as much as possible by (i) keeping the samples stored in a closed, clean box, away from light, (ii) changing the samples to new ones as often as possible, (iii) using fresh Milli-Q water in experiments, (iv) cleaning the acrylic tank after every day of experiments and (v) alternating the order of experiments with different samples. With this routine, we believe that surface contamination or bias from potential contamination could be successfully limited to a minimum, and be excluded as a mechanism responsible for the important wave attenuation presented in Fig. S7(d).

Due to incompatibility of the high speed imaging setup for observing the meniscus dynamics, the investigation of the influence of the meniscus dissipation on the wave attenuation, as suggested by Kidambi [9], is considered outside the scope of this work.

C. Details on the microfabrication process

The microfabrication process begins with the creation of a micro-patterned master, the shape of which is the negative of the desired geometry for the superhydrophobic sample. The master, which will be eventually used as a mold, is made of a photopolymer (SU-8 50, Micro Resist

Technology GmbH, Germany) deposited on a silicon wafer, spin-coated and finally baked. Using a maskless lithography instrument (MLA 150, Heidelberg Instruments GmbH, Germany), the coated wafer is then exposed to an ultraviolet (UV) laser beam (wavelength 375 nm), following a given pattern. The pattern layout (GDS II file format), generated with a mask design software (Klayout 0.27.2), mainly consists of a square lattice of circles with diameter of 20 µm and center-to-center spacing of 35, 40, 45, 55, 65, 75 or 85 µm. After UV exposure (dosage of 200 mJ cm^{-2} and defocus of 0), the wafer was baked and developed, revealing its microstructure, which is the negative of the desired design, *i.e.*, 20 µm wide circular pits arranged as described. The wafer is then put on a hot plate to bake (hard bake) for 20 minutes at 170°C. The last step of the master preparation consists in coating it with a thin layer of fluoropolymer via reaction-ion etching (Plasma RF generator, Oxford Instruments, United Kingdom). The fluorocompound CHF_3 (Trifluoromethane) is applied with a rate of 99.5 cm^3 per minute for 15 minutes, in a vacuum chamber with pressure of 250 mTorr (DC bias of 80 V).

PDMS (polydimethylsiloxane), the principal ingredient of the superhydrophobic surface, is prepared by mixing the monomer and the curing agent of Sylgard 184 (Dow Corning, Michigan, USA) in a 10:1 weight ratio. The obtained solution was degassed and then poured on the SU-8 wafer, previously treated with a fluoropolymer to facilitate the later peeling of the molded PDMS. The materials assembly was first left to degas again until all visible bubbles have disappeared and then baked in a hot-air oven for 3 hours at 80°C. The resulting cured PDMS cast was then gently peeled off from the mold before it underwent oxygen plasma for 10 minutes (Diener Electronic, Germany). A desiccator containing the PDMS sample and 100 µl of PFOTS (Trichloro(1*H*,1*H*,2*H*,2*H*-perfluorooctyl)silane, 97%, Sigma-Aldrich) was then placed in vacuum for chemical vapour deposition of PFOTS for 10 minutes. After that, the PFOTS was removed, so that only the PDMS sample was left for 30 more minutes in the same desiccator and vacuum system. At the end of these 30 minutes, the PDMS sample, having been rendered superhydrophobic by this process, was ready for experimentation.

The pillar height of the so-produced samples was tuned by changing the spinning speed, and the duration and temperature of the pre-bake and post-exposure bake, as detailed in Table S1. The pillar height was evaluated at two times during the process, once by measuring the pits depth of the micro-patterned wafer with a stylus profilometer (DektakXT, Bruker, USA) and once on the final sample using Scanning Electron Microscopy (Sigma VP, Zeiss AG, Germany), images of which are visible in Fig. S9(k-p).

D. Details on the plastron monitoring

The results on the plastron monitoring *via* the observation of the change of the phase speed with time, presented in Fig. 4, were produced with $h = 53$ µm. Another set of experiments conducted with $h = 21$ µm and with the same experimental conditions led to similar results, as illustrated in Fig. S8(b). The speed of the plastronic waves (i) increases as the plastron dissolves and (ii) decreases as the plastron inflates. It follows the same trend as in Fig. 4.

Accordingly to Fig. 2(b), this means that this effect is not solely governed by the variation of plastron thickness. Otherwise the trend in Fig. S8(b) would be reversed, compared to Fig. 4. Instead, the similarity of the results in both Fig. S8(b) and 4 suggest that a mechanism other than the plastron thickness is also involved and is directly driven by the inflation and the depletion of the plastron. Further studies would need to investigate the influence of the meniscus shape and the Laplace pressure, as these factors are known to be strongly linked to the wave frequency [9, 10].

E. Details on the image treatment and the analysis of wavefronts

A step-by-step exemplary case of the image treatment performed on the high-speed images is given in Fig. S10. Sequentially, the images are de-noised, convoluted by a vertical 3×3 kernel [-1 -1 -1; 2 2 2; -1 -1 -1], edge-highlighted and thresholded by area, so that the phase speed c_p can eventually be averaged from the angles of the detected wavefronts. A period of oscillation T is twice the average time step recorded every other wavefront, and the wavelength λ is calculated as $\lambda = c_p T$. In the exemplary case depicted in Fig. S10, the measured phase speed, frequency and wavelength are about 12 m s^{-1}, 10.4 kHz and 810 µm, respectively. As displayed in Fig. S1, this phase speed is approx. 16× the one of conventional capillary waves (in deep and shallow water) of similar wavelength, as computed from the theoretical dispersion relation of Eq. (1). The physics of these waves travelling on the plastron formed on a superhydrophobic surface must therefore considerably differ from conventional capillary waves. As discussed in the main document, that discrepancy could arise *e.g.*, from the presence of microstructures to which the

gas-water interface is coupled [11], but also to the mechanical impedance of the plastron, shifting in respect to its thickness.

For what concerns the evaluation of the wave attenuation, a transformation into a frequency-space domain is operated on the optical information expressed in time and space. Thereon, the first observation is the monochromatic character of the observed plastronic waves, as shown in Fig. S10(f) for the selected example. The wave attenuation is eventually assessed by scanning the frequency component fading out. The attenuation ratio α expressed in dB mm^{-1} is the one defined in the power law $e^{\alpha(f)r}$.

2. SUPPLEMENTARY FIGURES

Plastronic waves in the landscape of interfacial waves in deep and shallow water.

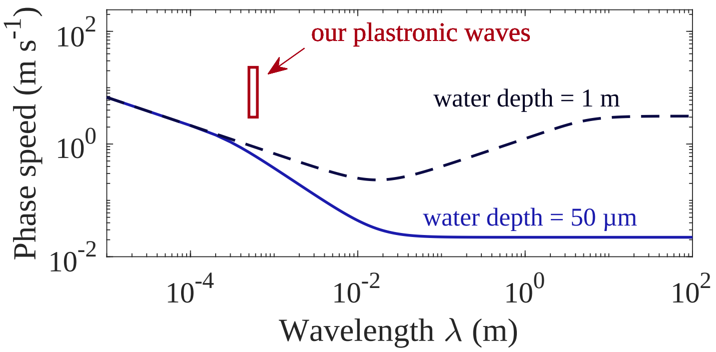

Fig. S1. Phase speed of interfacial waves in water of depth 1 m (dashed line) and 50 µm (solid line), as a function of the wavelength, in comparison with our experimental measurements of plastronic waves (red rectangle).

Summary of the investigated geometry of microstructures

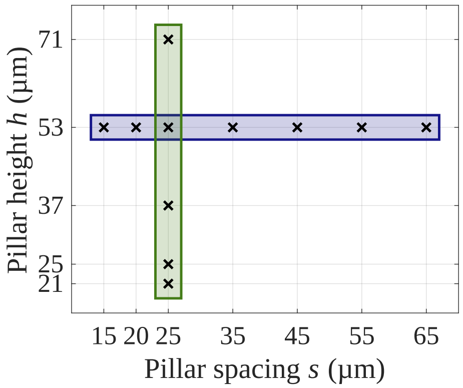

Fig. S2. Graphical summary of the configurations of microstructures employed in this work. While the pillar diameter is kept constant (20 µm), the investigated configurations differ by the pillar height h (21, 25, 37, 53 and 71 µm) or by the pillar spacing s (15, 20, 25, 35, 45, 55 and 65 µm), respectively with constant spacing $s = 25$ µm and height $h = 53$ µm.

Side-view demonstration of plastronic waves propagating on a gas-filled underwater microchannel

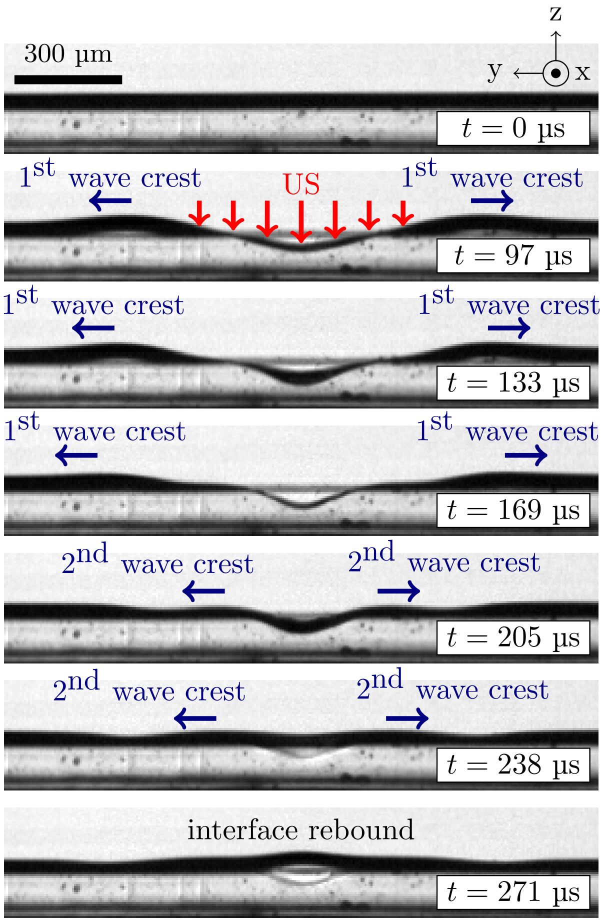

Fig. S3. The capability of Acoustic Radiation Force to induce a mechanical perturbation on a gas-water interface is demonstrated under high-speed imaging with the side view of a gas-filled microchannel submerged in water (long axis along y-axis, channel height = 100 µm and width = 75 µm), showing travelling interfacial waves propagating along the y-axis. For the generation of the interfacial wave, the US source is ON for a duration of 20 µs with pulse-average acoustic intensity $\simeq 14.1$ mW cm^{-2}.

Control on the frequency of the produced plastronic waves

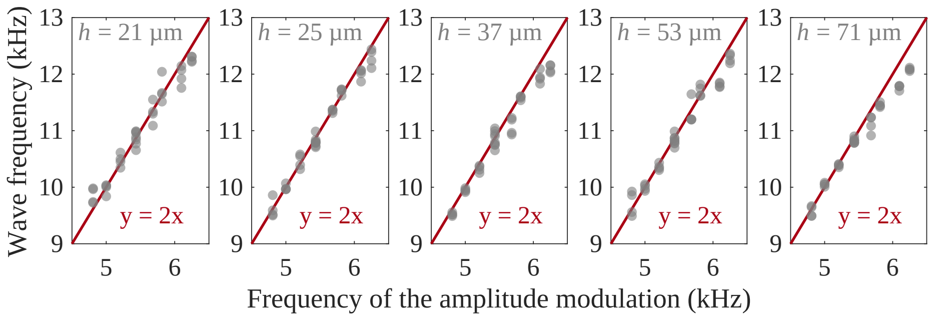

Fig. S4. The wave frequency as a function of the frequency of the amplitude modulation always exhibits a 2:1 ratio, regardless the pillar height, demonstrating the successful control on the frequency of the produced plastronic waves. Source data are provided as a Source Data file.

Frequency of the impulse response of plastron interface

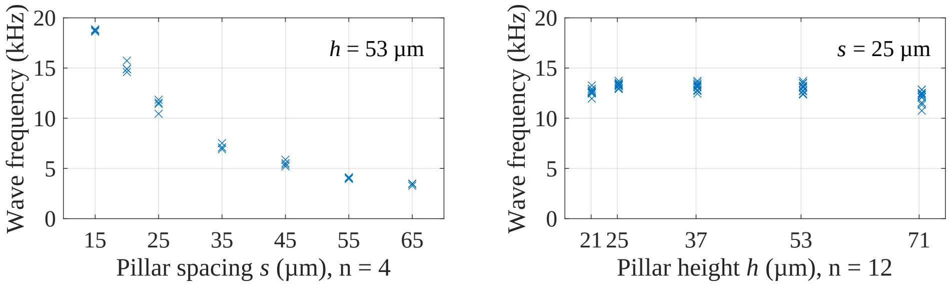

(a) (b)

Fig. S5. The natural frequency of the plastronic waves generated by a single US pulse (50 cycles, 2.5 MHz, no amplitude modulation) as a function of (a) the pillar spacing s (with constant $h = 53$ µm) or (b) the pillar height h (with constant $s = 25$ µm). These natural frequencies were used to define the central frequency of the range of amplitude-modulated frequencies employed to drive the plastron of each micropillar configuration. Source data are provided as a Source Data file.

Characteristics of the plastronic waves generated by a short US pulse (50 cycles, 2.5 MHz, not modulated in amplitude): wavelength-frequency analysis.

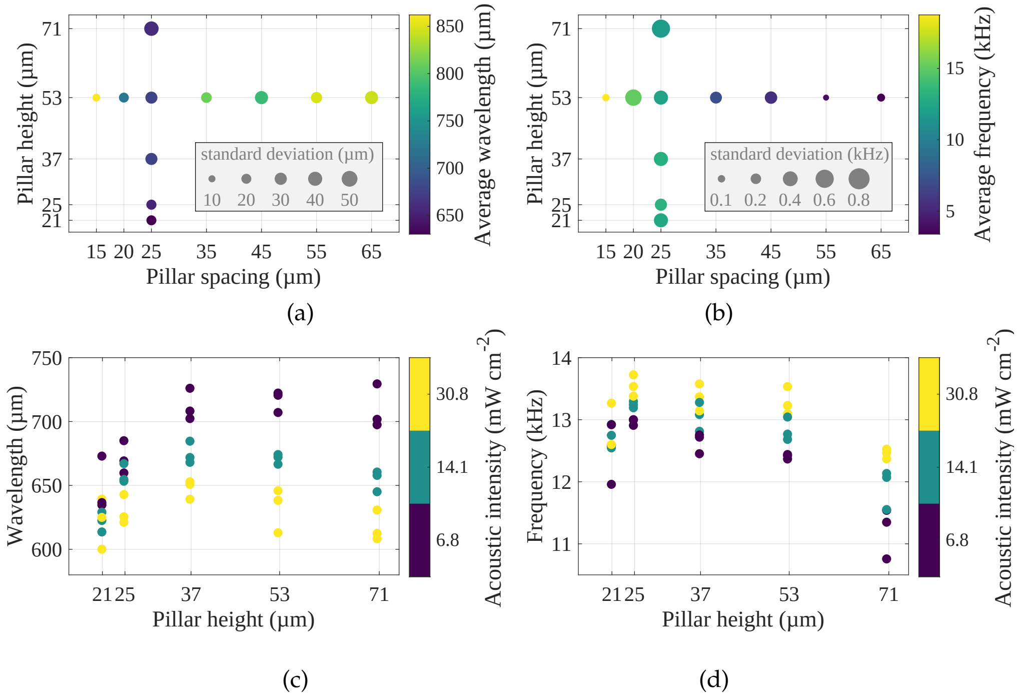

Fig. S6. Wavelength-frequency analysis, resulting from the tracking and analysis of capillary waves propagating on the plastron of a superhydrophobic surface. The average value and standard deviation of their wavelength (a) and frequency (b) are shown as a function of the pillar height and the pillar spacing, or solid-liquid contact area fraction. A detail of the wavelength (c) and frequency (d) as a function of the pillar height (pillar spacing = 25 µm) and the driving pulse-average acoustic intensity is provided. The latter is expressed in mW cm^{-2}. Source data are provided as a Source Data file.

Characteristics of the plastronic waves generated by a short US pulse (50 cycles, 2.5 MHz, not modulated in amplitude): phase speed and attenuation ratio.

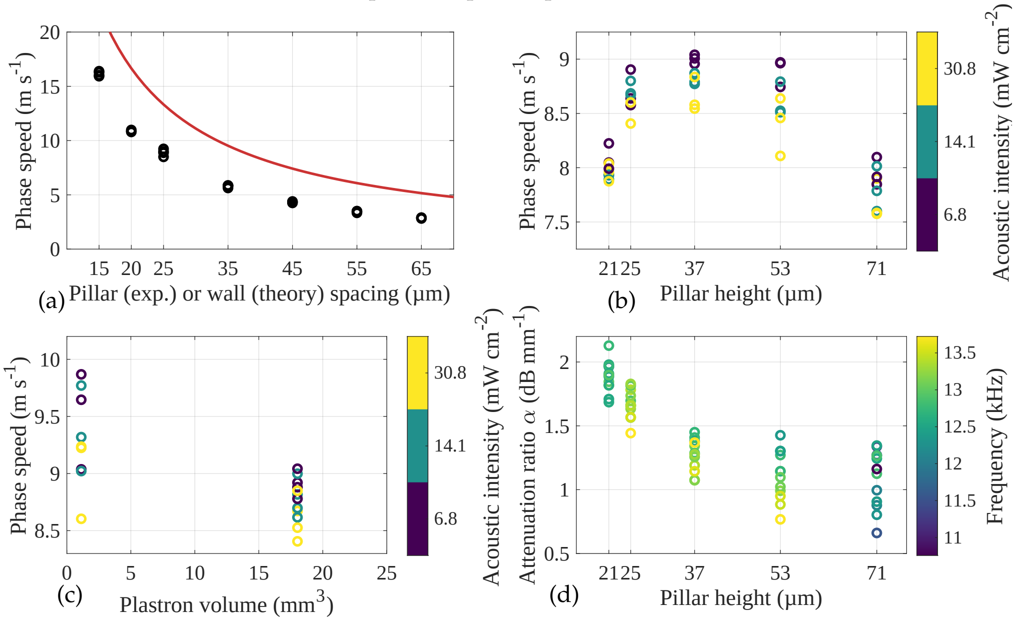

Fig. S7. Phase speed as a function of the pillar spacing (a), the pillar height (b) and the plastron volume (c), respectively, with fixed pillar height (53 µm), fixed pillar spacing (25 µm), and fixed pillar height (53 µm) and spacing (25 µm). For what concerns (a), our experimental results are compared with the semi-empirical law of Benjamin and Scott [11] drawn in solid red line, describing the phase speed of an interfacial wave ($\lambda = 800$ µm) travelling in a deep water-filled channel. To subfigures (b) and (c), the driving force expressed in mW cm^{-2} is informed. The attenuation ratio α of the same set of recorded waves is depicted in (d), all driving amplitudes combined. Source data are provided as a Source Data file.

Supplementary results on the monitoring of the plastron, via the analysis of the phase speed of plastronic waves shifting over time.

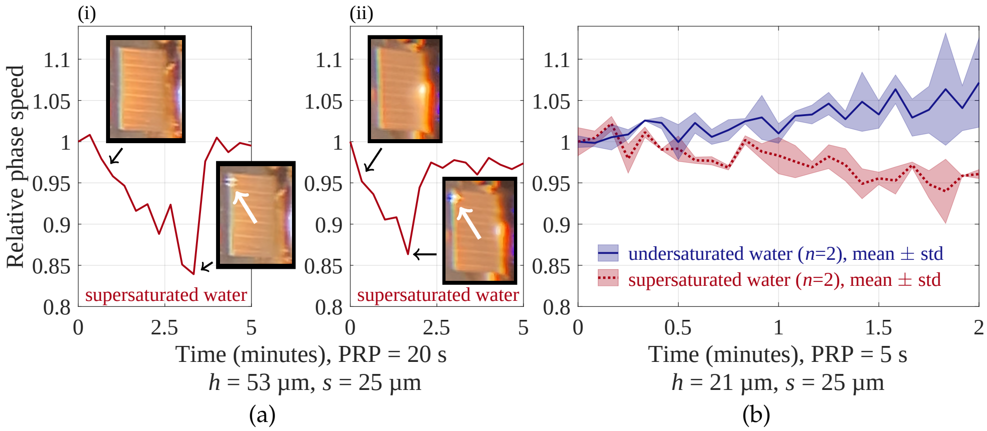

Fig. S8. (a) Two exemplary cases of plastron depinning, while the monitoring is ongoing. If the volume of gas in the plastron becomes too high due to important and fast spontaneous gas intake, the interfacial tensions holding the three-phase contact line may not hold. This happens here after (i) $\sim$ 3 minutes and (ii) $\sim$ 1.5 minutes. At that moment, both the formation of a bubble on the plastron (indicated by a white arrow) and a jump of the phase speed back towards its initial value are simultaneously observed. (b) With the same conditions of gas saturation as in Fig. 4, the monitoring of the plastron via the analysis of the phase speed was performed here, for the case of smaller pillar height (21 µm). A similar trend as in Fig. 4, *i.e.*, a slow-down of the waves as the plastron inflates (in red) and a speed-up of the waves as the plastron depletes (in blue) can be observed. The phase speed shift observed can be influenced by other factors than the thickening or the thinning of the plastron, requiring further studies. The parameter n refers to the number of repetitions for each experimental configuration. Source data are provided as a Source Data file.

Microfabrication steps and SEM images

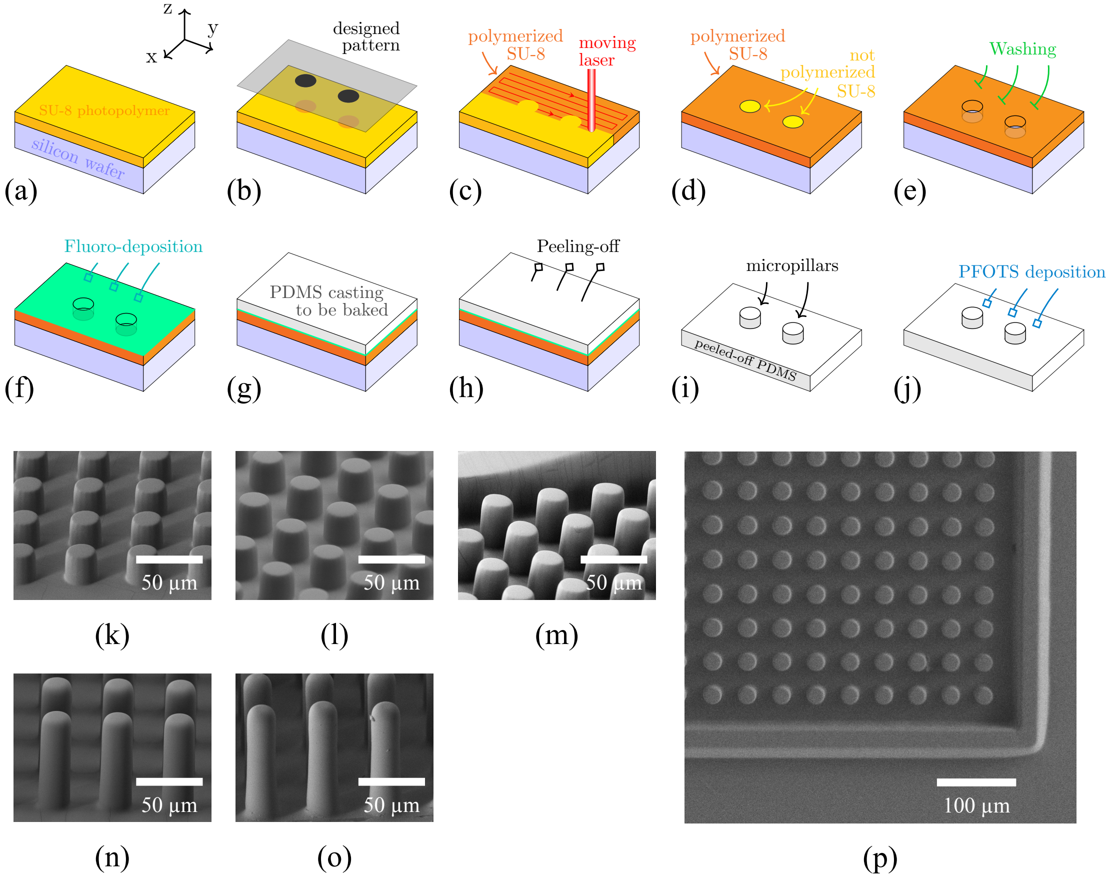

Fig. S9. Microfabrication steps process of the superhydrophobic surfaces. The master made on a silicon wafer (a) is spin-coated with a photosensitive polymer, which is SU-8. Tuning the height of the microstructures can be done by varying the rate of spinning, details in Table S1. Following a given design (b), a selective UV exposure of SU-8 is done using a UV laser beam (c), resulting in a pattern formed in SU-8 (d) resembling to micro-pits after washing (e). A fluoropolymer is deposited on the master to later enable an easy peeling of PDMS (f). PMDS is casted on the master and put to bake (g). The hardened casting is peeled off (h). Resulting PDMS micropillars (i). PFOTS deposition on PDMS micropillar arrays (j). The empirically-defined angle of UV exposure (365 nm, 200 mJ m^{-2}) and cooking time allow to give the micropillars the wanted geometry. Images obtained by Scanning Electron Microscopy (SEM) show examples of the so-produced different heights of micropillars, which are 21 µm (k), 25 µm (l), 37 µm (m), 53 µm (n) and 71 µm (o). A top view of the array edge and the surrounding wall is also depicted (p).

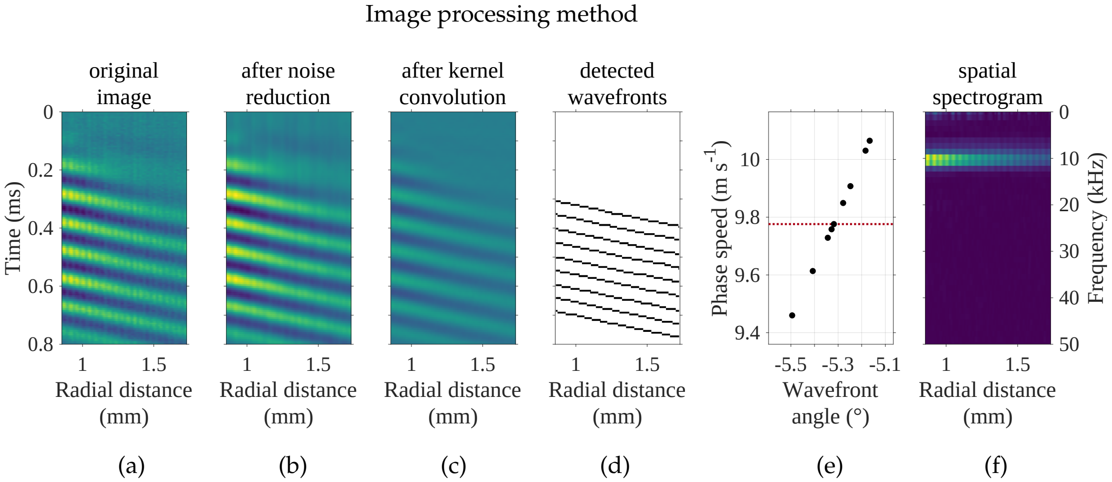

Fig. S10. Step-by-step image processing method. From left to right, the optical information expressed in polar coordinates (a) is sequentially de-noised (b), convoluted by a vertical kernel (c), edge-highlighted, and thresholded. The phase speed is eventually averaged from the angles of the obtained wavefronts (e). A fast Fourier transform applied on the time signals at each radial distance (f) allows to show that the wave frequency content is monochromatic (composed of one single frequency) and quickly dampens with radial distance r, following a power law $e^{\alpha(f)r}$. In this exemplary case, the frequency $f \simeq$ 10.4 kHz, the wavelength $\lambda \simeq$ 810 µm and the attenuation ratio $\alpha = 1.44$ dB mm^{-1}. The result suggests that the detected waves travel outwards from the point of actuation at a phase speed $c_\mathrm{p} \simeq$ 12 m s^{-1}, which is considerably faster ($> 16\times$) than the phase speed of conventional capillary waves with similar wavelength, travelling in deep water (c_p ∼0.75 m s^{-1}) and in shallow water (c_p ∼0.46 m s^{-1}).

Measurement method of the wave period and frequency

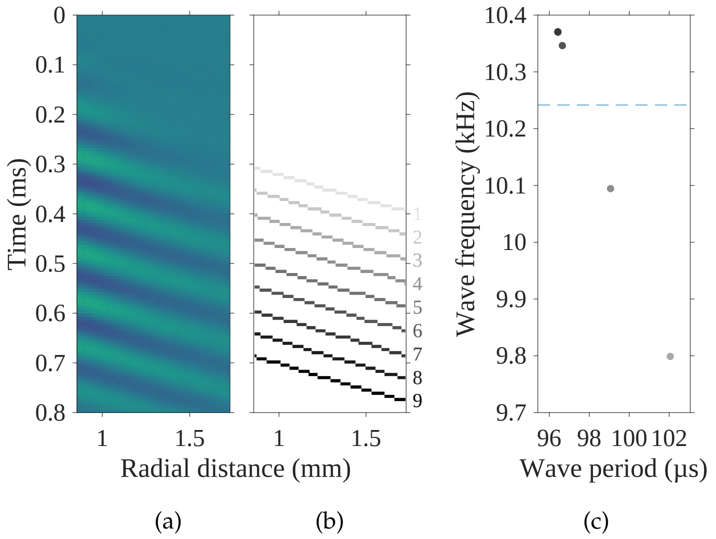

Fig. S11. The conversion of the high-speed video information into a polar coordinate system, the origin being the image-detected center of the acoustic actuation, allows to re-arrange the waves into a time *vs.* radial distance form, as exemplified in (a). After detection of the wavefronts (b), the wave period T is obtained by computing the average time spacing between wavefronts with same phase, *e.g.*, between the pair of wavefronts numbered 1 and 3. The operation is repeated for all pairs of detected wavefronts, such as numbered i and i+2. (c) The wave period T is eventually the mean of all values, and the wave frequency f is such that $f = 1/T$.

3. SUPPLEMENTARY TABLES

Preparation parameters of the superhydrophobic samples

pillar height	spinning speed for 30 s	pre-bake on hot plate	post-exposure bake on hot plate
21 µm	6200 rpm	65°C for 5 minutes<br>95°C for 8 minutes	65°C for 1 minutes<br>95°C for 8 minutes
25 µm	4000 rpm	65°C for 15 minutes<br>95°C for 20 minutes	65°C for 1 minutes<br>95°C for 10 minutes
37 µm	3000 rpm	65°C for 15 minutes<br>95°C for 20 minutes	65°C for 1 minutes<br>95°C for 10 minutes
53 µm	2250 rpm	65°C for 15 minutes<br>95°C for 25 minutes	65°C for 1 minutes<br>95°C for 10 minutes
71 µm	1750 rpm	65°C for 15 minutes<br>95°C for 25 minutes	65°C for 1 minutes<br>95°C for 10 minutes

Table S1. Summary of the preparation parameters of the superhydrophobic samples. The spinning speed, the pre-bake and the post-exposure bake are the decisive parameters for regulating the pillar height, measured afterwards using contact profilometry.

REFERENCES

1. B.-T. Chu and R. E. Apfel, "Acoustic radiation pressure produced by a beam of sound," The J. Acoust. Soc. Am. **72**, 1673–1687 (1982).
2. G. Kim, S. Cheng, L. Hong, *et al.*, "On the acoustic fountain types and flow induced with focused ultrasound," J. Fluid Mech. **909** (2021).
3. L. Krutyansky, A. Brysev, F. Zoueshtiagh, *et al.*, "Measurements of interfacial tension coefficient using excitation of progressive capillary waves by radiation pressure of ultrasound in microgravity," Microgravity Sci. Technol. **31**, 723–732 (2019).
4. N. L. Walbridge and L. A. Woodward, "Phase velocity of surface capillary-gravity waves," Phys. Fluids **13**, 2461 (1970).
5. D. Bartolo, F. Bouamrirene, E. Verneuil, *et al.*, "Bouncing or sticky droplets: Impalement transitions on superhydrophobic micropatterned surfaces," Europhys. Lett. **74**, 299–305 (2006).
6. M. J. Hokkanen, M. Backholm, M. Vuckovac, *et al.*, "Force-based wetting characterization of stochastic superhydrophobic coatings at nanonewton sensitivity," Adv. Mater. **33**, 2105130 (2021).
7. S. Herminghaus, M. Brinkmann, and R. Seemann, "Wetting and dewetting of complex surface geometries," Annu. Rev. Mater. Res. **38**, 101–121 (2008).
8. F. Shan, J. Xiao, Z. Chai, and B. Shi, "Pinning and depinning in imbibition beyond a sharp edge: A lattice boltzmann study," Int. J. Multiph. Flow **159**, 104317 (2023).
9. R. Kidambi, "Meniscus effects on the frequency and damping of capillary-gravity waves in a brimful circular cylinder," Wave Motion **46**, 144–154 (2009).
10. P. N. Shankar, "Frequencies of gravity–capillary waves on highly curved interfaces with edge constraints," Fluid Dyn. Res. **39**, 457–474 (2007).
11. T. B. Benjamin and J. C. Scott, "Gravity-capillary waves with edge constraints," J. Fluid Mech. **92**, 241–267 (1979).
