# Supplementary material for: Fast capillary waves on an underwater superhydrophobic surface
Source: Nat Commun. 2025 Feb 12;16:1568. doi: 10.1038/s41467-025-55907-w (PMC11821838; doi:10.1038/s41467-025-55907-w)
Supplement: Supplementary file 2 — Description of Additional Supplementary Files [file 41467_2025_55907_MOESM2_ESM.pdf]

NCOMMS-24-02897C: Description of Additional Supplementary Files

Caption of Movie 1:

Exemplary video of circular plastronic waves travelling on the gas-water interface of the plastron of an underwater superhydrophobic surface, patterned with micropillars of height 53 µm, radius 10 µm and spacing 25 µm. The 2.5 MHz ultrasound driving signal is modulated in amplitude with a frequency $f_{\mathrm{AM}} = 6098$ Hz and lasts about $\frac{3}{f_{\mathrm{AM}}} \simeq 492$ µs. The recording and playback frame rates being, respectively, 159 090 and 20 images per second, means that this video is accelerated by 7950, approximately. The only image processing that was applied to this video is a background (first image) subtraction. In this example, which correspond to the provided Supplementary dataset and algorithm, the plastronic waves have a propagation speed of ~ 9.2 m s^{-1}, a frequency of 12.1 kHz and a wavelength of 760 µm.

NCOMMS-24-02897C: Description of Additional Supplementary Files

File name: Supplementary Data 1
Description: Home-made algorithm developed in Matlab language. It analyses a high-speed video of circular plastronic waves, induced by an amplitude-modulated focused ultrasound pulse. The algorithm takes as an input a high-speed video in .mat format, such as the dataset provided in Supplementary Data 2. After a detection of the wavefronts, the algorithm computes the wave characteristics, i.e., phase speed, wavelength, frequency and attenuation ratio.

File name: Supplementary Data 2
Description: Exemplary dataset (.mat file) of circular plastronic waves travelling on the gas-water interface of the plastron of an underwater superhydrophobic surface, patterned with micropillars of height 53 µm, radius 10 µm and spacing 25 µm. The 2.5 MHz ultrasound driving signal is modulated in amplitude with a frequency $f_{\mathrm{AM}} = 6098$ Hz and lasts about $\frac{3}{f_{\mathrm{AM}}} \simeq 492$ µs. In this example, the plastronic waves have a propagation speed of $\sim$9.2 m s^{-1}, a frequency of 12.1 kHz and a wavelength of 760 µm.
