# Supplementary figures and images for: Fast capillary waves on an underwater superhydrophobic surface
Source: Nat Commun. 2025 Feb 12;16:1568. doi: 10.1038/s41467-025-55907-w (PMC11821838; doi:10.1038/s41467-025-55907-w)

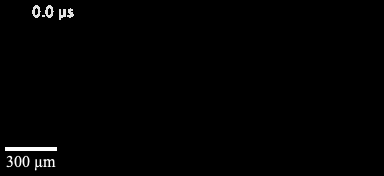

Supplement: Supplementary file 3 — Movie 1 [file 41467_2025_55907_MOESM3_ESM.gif]
